# Enhancing Water Treatment Performance of Porous Polysulfone Hollow Fiber Membranes through Atomic Layer Deposition

**DOI:** 10.3390/molecules28166133

**Published:** 2023-08-18

**Authors:** Jeanne Casetta, Céline Pochat-Bohatier, David Cornu, Mikhael Bechelany, Philippe Miele

**Affiliations:** 1 Institut Européen des Membranes—IEM, UMR-5635, University of Montpellier, ENSCM, CNRS, Place Eugene Bataillon, 34095 Montpellier, France; jeanne.casetta@umontpellier.fr (J.C.); david.cornu@umontpellier.fr (D.C.); philippe.miele@umontpellier.fr (P.M.); 2 Applied Mathematics and Bioinformatics (CAMB), Gulf University for Science and Technology—GUST, Kuwait City 32093, Kuwait

**Keywords:** membrane, hollow fibers, atomic layer deposition, molecular layer deposition, polysulfone

## Abstract

Polysulfone (PSF) is one of the most used polymers for water treatment membranes, but its intrinsic hydrophobicity can be detrimental to the membranes’ performances. By modifying a membrane’s surface, it is possible to adapt its physicochemical properties and thus tune the membrane’s hydrophilicity or porosity, which can achieve improved permeability and antifouling efficiency. Atomic layer deposition (ALD) stands as a distinctive technology offering exceedingly even and uniform layers of coatings, like oxides that cover the surfaces of objects with three-dimensional (3D) shapes, porous structures, and particles. In the context of this study, the focus was on titanium dioxide (TiO2), zinc oxide (ZnO), and alumina (Al_2_O_3_), which were deposited on polysulfone hollow fiber (HF) membranes via ALD using TiCl_4_, diethyl zinc (DEZ), and trimethylamine (TMA), respectively, and H_2_O as precursors. The morphology and mechanical properties of membranes were changed without damaging their performances. The deposition was confirmed mainly by energy-dispersive X-ray spectroscopy (EDX). All depositions offered great performances with a maintained permeability and BSA retention and a 20 to 40° lower water contact angle (WCA) than the raw PSF HF membrane. The deposition of TiO_2_ offered the best results, showing an enhancement of 50% for the water permeability and 20% for the fouling resistance of the PSF HF membranes.

## 1. Introduction

Among all polymers, polysulfone (PSF) is widely chosen for membrane applications and especially hollow fiber (HF) membrane fabrication due to its remarkable chemical and thermal resistance, which makes it suitable for membrane washing and ensuring long-term usage with good mechanical properties [1,2]. However, the inherent hydrophobicity of this polymer affects a membrane’s performances especially in water treatment applications because of contaminant fouling on its surface. That is why surface modification of these hydrophobic polymeric membranes appears to be an interesting way to overcome this issue and thus enhance both the membranes’ water permeability and resistance to fouling [3,4]. Coating methods offer a viable solution for surface modification involving the deposition of a thin layer of material onto the membrane surface. Various techniques [5], such as sol-gel, physical vapor deposition (PVD), chemical vapor deposition (CVD), or atomic layer deposition (ALD), can be employed for this purpose. For instance, Zhang et al. prepared and characterized a PSF-TiO_2_ HF ultrafiltration membrane using the sol–gel method. With only 2 wt. % TiO_2_, they observed a water contact angle (WCA) that was significantly reduced from 85° to 55° and a maximum of 96.3% bovine serum albumin (BSA) rejection [6]. While all of these methods show promise and deliver interesting results, ALD stands out due to its exceptional surface conformality. Unlike PVD, which only affects the surface; sol-gel, which can block pores; and CVD, which may not provide uniform coating throughout, ALD enables the deposition of highly uniform coatings over three-dimensional (3D) surfaces, deep trenches, porous materials, and particles thanks to its surface-controlled film growth [7]. ALD is a relatively recent technique developed over the past four decades that allows for the deposition of precise sub-nanometer-thickness coatings of various inorganic materials. This method has already attracted interest for water treatment applications. Yang et al. laid out a pathway to establishing an ALD-based universal functionalization platform for water treatment and detailed a numerous of water applications for ALD-modified materials [8].

Recent studies have demonstrated the applicability of ALD to flexible and temperature-sensitive polymeric membranes [9,10]. Deposition of an oxide thin layer through ALD has been shown to enhance membrane hydrophilicity, modify the pore size [11], and reduce membrane fouling, albeit at the expense of water permeability. Wang et al. deposited polyimide on the surface of nanoporous anodized alumina using ALD. They found that 50 cycles resulted in a remarkable improvement in retention, increasing it from nearly none for the neat alumina substrate to 82% while maintaining a high water permeability of 800 L/hm^2^bar, albeit about three times lower compared to the uncoated membrane [12]. Feng et al. ALD-deposited ZnO on fabrics of multi-walled carbon nanotubes. ZnO was grown on the CNT surface as nanoparticles initially; it then formed conformal layers wrapping the CNTs. The originally hydrophobic surface of the CNTs was progressively tuned to be highly hydrophilic with rising ALD cycles, and the WCA was reduced from 110° to almost 20° after 100 ALD cycles. They also observed a six-fold increase in BSA rejection with the highest permeability value of 430 L/hm^2^bar after 40 ALD cycles [13]. Jia et al. proved that ALD was an effective strategy to conveniently upgrade the filtration performances of polypropylene HF (PPHF) membranes by studying the deposition of Al_2_O_3_. In fact, they showed simultaneously enhanced water permeability and retention after deposition with moderate ALD cycle numbers. After 50 ALD cycles, they obtained a 17% increase in water permeability and a one-fold increase in BSA rejection accompanied with an improved fouling resistance for the modified PPHF [14]. Nikkola et al. presented the surface modification of a thin-film composite (TFC) polyamide (PA) reverse osmosis (RO) membrane via ALD using trimethyl aluminum (AlMe_3_) in order to improve its antifouling performances. The most hydrophilic surface was obtained with 10 and 50 ALD cycles at a temperature of 70 °C and 10 ALD cycles at 100 °C, respectively leading to a 16° and 27° WCA compared to the initial 53°. The antifouling properties of the membranes were also improved [15]. Li et al. focused on the hydrophilic modification of polyvinylidene fluoride (PVDF) membranes via ZnO ALD using nitrogen dioxide and diethylzinc functionalization. In this way, they attempted to counter the granular deposits that can sometimes sacrifice membrane flux even if they enhance hydrophilicity. They observed that after 200 ALD cycles, the WCA decreased from 140° of the original membrane to 67° and 20° for the direct modified membrane and the NO_2_/DEZ activated membrane, respectively. In addition, compared with direct modified membranes, a maximum water flux of 5261.4 L/hm^2^bar and a superior separation performance with 97% BSA retention were achieved for activated membranes at only 100 ALD cycles [16]. Wang et al. studied TiO_2_ deposition on PVDF membranes. They explained that the deposition of TiO_2_ enhanced the hydrophilicity and fouling resistance of the modified membranes, which was more evident at higher ALD cycle numbers. They simultaneously achieved an almost three-fold improved water flux and retention at 120 ALD cycles. This was as a result of the competing effect of the increased hydrophilicity from 67° to 30° and reduced pore sizes. They added that the water flux could be sensitively tuned by changing the exposure time of the precursors [17].

For the first time, this paper focused on the deposition of three different oxides on PSF HF membranes and their interest for water treatment applications. In a previous work, the impact of 10 to 100 cycles of TiO_2_ ALD on PSF HF membranes was investigated. The results were encouraging, as we obtained increases of 50% in water permeability and 20% in fouling resistance for a thickness deposition of only 2 nm [18]. In this study, the focus was on comparing the deposition of Al_2_O_3_, ZnO, and TiO_2_ using ALD on PSF HF membranes. The effect of ALD treatment on membrane morphology was investigated using scanning electron microscopy (SEM), energy-dispersive X-ray spectroscopy analysis (EDX), and water contact angle (WCA) measurements to assess hydrophilicity. The performance of the HF membranes for water-purification applications was characterized in terms of water permeability, antifouling properties using bovine serum albumin (BSA) protein, and mechanical properties. Prior to ALD treatment, the polymeric membranes underwent a conditioning step to ensure their ability to withstand vacuum and heat treatment. Based on our previous study [18] and the best results obtained for a 2 nm TiO_2_ deposition, all membranes were placed in the deposition chamber with glycerin conditioning while aiming for a targeted thickness of 2 nm.

## 2. Results and Discussion

### 2.1. Structural, Physical, and Chemical Characterizations

The formation of oxide layers on the PSF HF membranes was investigated via ATR-FTIR spectroscopy. In Figure S1, the characteristic peaks of PSF are visible in the raw and modified membranes. Signals observed around 1100, 1490, 1510, and 1590 cm^−1^ corresponded to the vibrations of PSF aromatic rings (C=C stretching). Bands at 1150 cm^−1^ and 1240 cm^−1^ were attributed to the –O–S–O– symmetric and aromatic ether bond (–C–O–C–), respectively. The peak at 1295 cm^−1^ was related to SO_2_ group stretching vibration [19]. Additionally, the absorbance peak around 1680 cm^−1^ could be attributed either to the C=O stretching vibration of NMP or PVP that might remain on the membrane surface. For modified membranes, any new peaks could be observed in the investigated frequency range. Thus, we can assume that the amount of oxides of around 2 nm thickness was too low to be detected via this technique as a consequence of a low number of ALD cycles. We expected to observe the metal oxide interaction peak with the PSF HF membranes. For example, for Al, we could have seen the Al-O peak that would appeared around 700 cm^−1^ [14]. In the case of Zn, two peaks could be observed between 500 and 630 cm^−1^ corresponding to the Zn-O bond [20]. Jia et al. studied the deposition of Al_2_O_3_ on porous polypropylene HF via the ALD technique, and they only noticed a new absorption peak after more than 100 deposition cycles [14].

**Figure 1:**
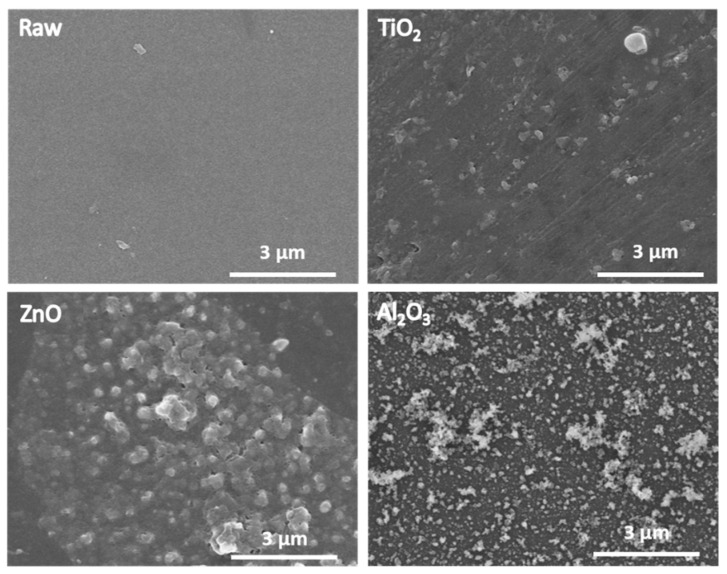
SEM surface images of raw and TiO_2_-, ZnO-, and Al_2_O_3_-modified PSF HF membranes.

SEM analyses performed on the top surface of a raw PSF HF membrane and modified PSF HF membranes are presented in Figure 1. No noticeable change was observed on the surface of oxide-deposited membranes. In the case of the ZnO- and Al_2_O_3_-modified membranes, some aggregates were observable on the surface. However, the number of cycles used in the deposition process was relatively low, making it challenging to clearly determine the oxide thickness via SEM. For comprehensive visualization, primary cross-section and top-surface SEM images of all membranes are provided in Supplementary Figure S2.

**Figure 2:**
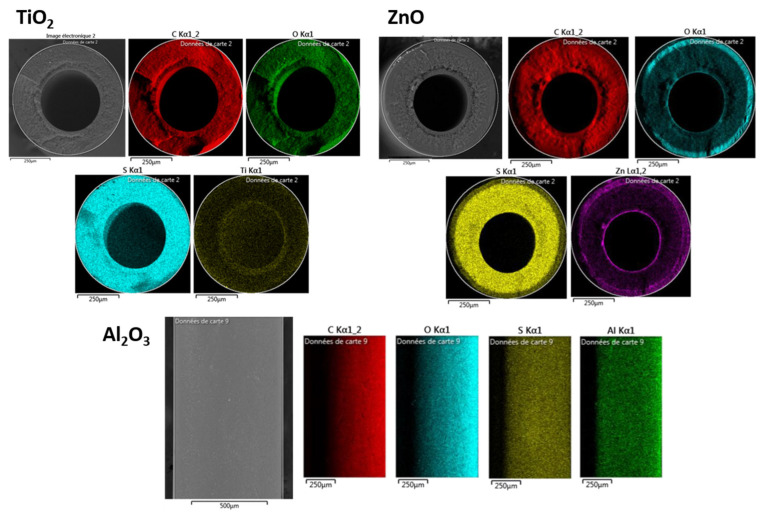
EDX mapping of TiO_2,_ ZnO (cross section), and Al_2_O_3_ (surface) modified PSF HF membranes.

In order to confirm the oxide deposition and attempt to quantify the amount deposited on the membranes, EDX of all raw and modified PSF HF membranes was performed. The measured atomic percentages of C, O, and Ti are presented in Table 1. The detected atomic percentages of Ti were very low due to the small layer thicknesses of the oxides. However, for the ZnO- and Al_2_O_3_-modified membranes, the atomic percentages of Zn and Al respectively attained 1.7 and 3.2% and displayed a much higher O percentage (34.3 and 47.8%, respectively) compared to the raw PSF HF membrane with 9.9%. This confirmed the deposition of ZnO and Al_2_O_3_ on the entire membrane surface. There are two plausible explanations for the significant difference in titanium content compared to zinc and aluminum in the sample. Firstly, the EDX method used for analysis may exhibit varying detection efficiency for different elements. Moreover, the detection may be affected when a peak appears alone or overlaps with other peaks. In the case of titanium, it seemed to appear at the same energy (keV) as oxygen, which could contribute to its reduced detection. Secondly, the variation in precursor materials and their interactions with the substrate plays a crucial role. Factors like steric hindrance and diffusion can differ depending on the specific precursor used, leading to variable diffusion and deposition within the pores of the membranes as well as within the polymeric chain of the membranes. Consequently, certain oxides, such as titanium in our case, may not be detected as effectively as the others. In addition, EDX surface and cross-sectioned mapping were also investigated; these are presented in Figure 2. All modified membranes demonstrated a uniform deposition of the oxide on the surface, but only TiO_2_ and ZnO also had a great diffusion of the oxide onto the membrane network as suggested by the cross-sectioned images. These results could be optimized by modifying the deposition conditions of Al_2_O_3_.

Due to the considerably low Ti atomic percentages observed in the EDX analysis, XPS analyses were carried out in a previous study [18]. For the 20-cycle samples, the results were once again below the detection limit, but when using 50 cycles, the presence of Ti was clearly evident in the XPS analysis. The XPS survey spectra of both the raw and TiO_2_ 50-cycle modified PSF HF membranes are presented in Figure S3. In both cases, the survey spectra showed two peaks at 284.85 and 532.05 eV, which were assigned to C and O atoms, respectively. Additionally, a new peak appeared at 458.36 eV in the ALD-modified PSF HF membrane, corresponding to Ti atoms. The C 1s spectra for both membranes were similar, indicating that the PSF HF membrane remained undamaged during deposition. The deconvoluted O 1s spectra showed signals for O-C and O=S bonds, and a new peak at 529.86 eV was observed for the modified membrane, indicating the presence of a Ti-O bond. This confirmed the successful deposition of TiO_2_, which reacted with the oxygen atoms of the PSF matrix. Moreover, in the deconvoluted Ti 2p spectra (Figure S3f) for the ALD-modified PSF HF membrane, two peaks were identified at 458.36 and 464.16 eV, corresponding to Ti 2p3/2 and Ti 2p1/2 states, respectively, indicating that Ti existed in the 4^+^ valence state. In this work, XPS analyses were not conducted on the ZnO- and Al_2_O_3_-modifed PSF HF membranes, but we trusted a previous study of similar deposition investigated via XPS. In fact, Viter et al. prepared ZnO/Al_2_O_3_ nanolaminate coated electrospun nanofibers and were able to confirm the presence of Zn and Al from the deposition via XPS. In addition, it was confirmed that no hydrated oxides were formed on the nanofibers’ surfaces [21].

### 2.2. Membrane Performances

**Figure 3:**
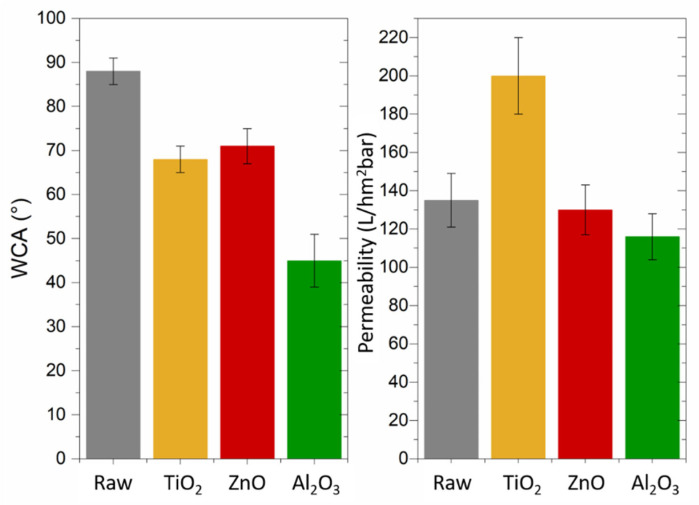
WCA and water permeability of raw and TiO_2_-, ZnO-, and Al_2_O_3_-modified membranes. For water permeability, the error refers to the standard deviation of 3 samples. In the case of the WCA, the contact angle was measured 3 times on each droplet image, and this was repeated on 5 to 10 samples.

Mechanical tests were performed on raw and modified membranes; the Young’s modulus, strength at break, and elongation at break values are presented in Table 2. The results showed that the modified membranes displayed a higher Young’s modulus of around 150 MPa on average compared to the raw membrane (132 MPa), which corresponded to a lower resistance to stress deformation. Because the ALD-modified membranes were more rigid, the strength applied to break them was lower than for the raw PSF HF membrane, decreasing from 2.10 ± 0.02 to 1.51 N on average. Also, the elongation at break was increased for the TiO_2_-modified membranes, whereas it remained stable or decreased for the other ones. Tensile strength curves for all membranes are presented in Figure S5. Nevertheless, TiO_2_ might induce a nanocomposite impact within the polymer chains, thereby enhancing the overall robustness of the network structure and consequently bolstering its mechanical resilience [22]. This observation aligns with the findings of Diez-Pascual et al., who investigated the impact of introducing TiO_2_ nanoparticles into polyphenylsulfone. They documented a progressive increase in the Young’s modulus as the nanoparticle loading was raised, reaching up to a 62% enhancement at the highest concentration evaluated. This enhancement was attributed to the influence of the inflexible TiO_2_ nanoparticles, which established interactions (such as H-bonding) with the membrane matrix. These interactions led to a reduction in polymer chain mobility and facilitated stronger adhesion between the two phases [23]. Figure 3 provides the permeability data, which indicated a notable enhancement in the water permeability for the TiO_2_-modified membranes, while the permeability values remained consistent for the membranes modified with ZnO and Al_2_O_3_. According to the mechanical properties and permeability and compared to other oxides, TiO_2_ seemed to be the best candidate for improving the PSF HF membranes’ properties. Interestingly, Al_2_O_3_ deposition showed differences in terms of mechanical properties compared to the other modified membranes. One hypothesis could be that Al_2_O_3_ spread between polymer chains and not the pores, causing polymer chain infiltration and thus alteration of the mechanical properties; this would be in good agreement with EDX analysis above.

To complete the experiment, the WCAs were measured for the raw and modified membranes, and the results are presented along with the water permeability values in Figure 3. Initially, the raw membrane had a WCA of around 90°, and this value was gradually reduced down to 70° with TiO_2_ deposition. These findings aligned well with the existing literature. For instance, Zhang et al. fabricated a hybrid ultrafiltration flat membrane by incorporating TiO2-g-HEMA and examined its resistance to fouling. They achieved the most substantial enhancement in hydrophilicity marked by a water contact angle (WCA) of 72° at a concentration of 2%, as the study reported [24]. The WCA was also reduced to 70° and even to 45° with ZnO and Al_2_O_3_ oxides, respectively, accompanied by a stable water permeability compared to the raw membrane. Li et al. studied the hydrophilic modification of polyvinylidene fluoride membranes via ZnO atomic layer deposition and observed a stable water flux and a WCA reduction only above 50 cycles [16]. Lin et al. prepared Al_2_O_3_/PES composite hollow fiber UF membranes via facile in situ vapor-induced hydrolyzation and observed a WCA reduced from 80° to 40° but with almost twice the reduced water permeability [25]. Once again, TiO_2_ seemed to be the best oxide candidate for improving the PSF HF membranes properties by combining a lower WCA and higher water permeability. A previous study thoroughly investigated and ruled out the possibility that the superior performances of TiO_2_-modified PSF HF membranes were solely attributed to the thermal conditions of the ALD process [18]. In Figure S4, SEM images and permeability values are presented for a comparison between the TiO_2_-modified PSF HF membrane and a membrane that underwent the same vacuum and temperature process for the same duration but without the deposition. The membrane without deposition displayed a permeability of only 27 L/hm^2^bar, whereas the ALD-modified PSF HF membrane exhibited a significantly higher permeability of 200 L/hm^2^bar. These results provided strong evidence that the outstanding performances of TiO_2_-modified membranes cannot be solely attributed to the thermal parameters of the ALD process.

**Figure 4:**
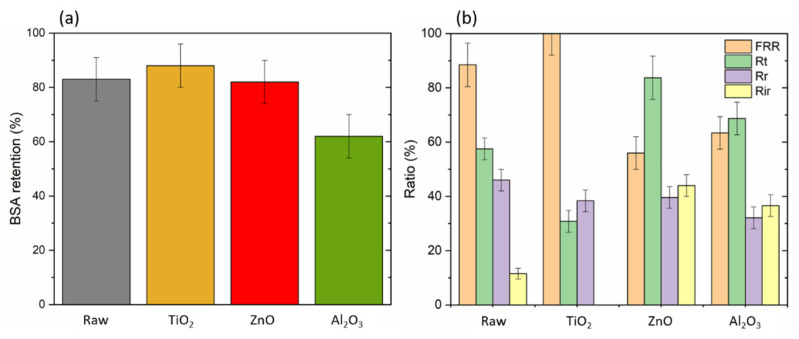
(**a**) BSA retention and (**b**) fouling resistance parameters for raw and TiO_2_-, ZnO-, and Al_2_O_3_-modified membranes (the error refers to the standard deviation of 3 samples).

The filtration performances of the raw and modified PSF HF membranes are depicted in Figure 3 and Figure 4a. The raw membrane exhibited a pure water permeability of 135 L·h^−1^m^−2^bar^−1^ and a retention of BSA of around 80%. For all modified membranes, the BSA retention rate was quite higher or stable except for that with the Al_2_O_3_ deposition, which decreased to 60%. Antifouling performances of the modified membranes were investigated using BSA as a fouling agent; for this, the FRR, $R_{t}$, $R_{r},\;$ and $R_{ir}$ of the membranes were calculated and are presented in Figure 4b. The FRR was increased from 88% for the raw PSF HF membrane to 100% only for TiO_2_, whereas it decreased to 60% on average for all other oxides. Following this trend, once again only the TiO_2_-modified membrane exhibited a lower $R_{t}$, $R_{r},\;$ and $R_{ir}$. A decreasing $R_{r}\;$ and $R_{ir}$ means that BSA adsorbed reversibly was removed after water backwashing and none of it remained, which was a good result because membrane lifetimes are threatened by fouling and most of all irreversible fouling, and chemical backwashing is needed to clean them. This result means that due to TiO_2_ addition, the ALD-modified PSF HF membranes were recovering all the flux after BSA filtration with no irreversible fouling. These results are very interesting since the values are even better than those reported in other studies. Yang et al. [26] and Zhang et al. [24] inserted, for instance, TiO_2_ nanoparticles into flat PSF membranes and obtained 97 and 95% BSA rejection, respectively. In fact, BSA is a complex protein of 67kDa, the behavior of which is widely influenced by its environment. Several studies focused on this understanding and can provide some information to comprehend the adsorption mechanism of the protein onto the substrate [27,28,29]. BSA can be adsorbed either by electrostatic or hydrophobic interactions, meaning that both the WCA and zeta potential of the membranes are key parameters. The isoelectric point of a protein plays an important role in transport because it determines the surface charge of the protein, depending on the pH of the solution. When the pH value of a buffer solution is equal to the isoelectric point of the protein, the surface charge of the protein is neutral; at a pH above that point, the protein is negatively charged (and vice versa at a lower pH) [29]. So, BSA has an isoelectric point of 4.7, which means that at the experimental pH of 7.4, its surface is negatively charged. In the case of TiO_2_, multiple works have shown that adding it to a polymeric membrane would reinforce the negative charge of the surface, enhancing the repulsive interactions between the membrane and BSA [30,31,32]. TiO_2_ both improved the hydrophilicity and negatively charged surface of the PSF HF membranes with an initial zeta potential known to be around −10 mV [33], which explains the great BSA retention as well as the high fouling parameters because it was widely spread onto the matrix as proved by EDX observations. TiO_2_, ZnO, and Al_2_O_3_ should have the save behavior in terms of zeta potential at around pH 7.4 [34]. Huang et al. incorporated oleic acid-modified Ag@ZnO core–shell nanoparticles into thin-film composite membranes and observed enhanced antifouling properties against BSA. They showed that ZnO could increase the membrane’s negative surface charge or slowly reduce it, depending on the proportion of nanoparticles [35]. In our case, the ZnO-modified membrane had the same WCA properties as the TiO_2_ one, but the negative surface charge possibly was not enough to repulse the BSA and prevent it from entering the polymer matrix, causing lower fouling parameters. Concerning Al_2_O_3_, Jia et al. deposited Al_2_O_3_ on PP HF membranes and obtained a maximum of 75% BSA rejection [14]. We can suppose than despite the highly reduced WCA of the Al_2_O_3_-modified membrane, the surface charge and roughness, which could not be measured in the study, were too high to allow better antifouling properties against BSA compared to the raw PSF HF membrane. Also, we utilized fouling data to confirm the stability of the membrane. The data demonstrated that the modified membrane retained the same performance even after several hours of filtration, which validated the presence and maintenance of the oxide layer on the surface of our support.

## 3. Materials and Methods

### 3.1. Materials

This study utilized PSF hollow fiber (PSF HF) membranes supplied by Polymem (Fort Worth, TX, USA) as the substrates for atomic layer deposition (ALD). The membranes underwent chlorine washing and glycerin conditioning steps before the deposition process. The following materials were employed for the ALD process: titanium (IV) chloride (TiCl_4_, 99.9%, CAS: 7550-45-0), diethylzinc (DEZ, (C_2_H_5_)2Zn, >95%, CAS: 93-3030), trimethylaluminum (TMA, Al(CH_3_)_3_, >98%, CAS: 93-1360), and deionized water. Argon gas, which was purchased from Linde (Dublin, Ireland), was utilized as received. Phosphate-buffered saline (PBS) from Roth (Karlsruhe, Germany) and bovine serum albumin (BSA) solution with a molecular weight of 67 kDa and a purity of ≥96% from Sigma-Aldrich (St. Louis, MO, USA) were used to evaluate the fouling resistance of both the untreated and modified PSF HF membranes. Deionized water from Millipore Milli-Q (Burlington, MA, USA) was used in all aqueous solutions throughout the experiment.

### 3.2. Oxide Deposition on PSF HF Membranes

The commercial PSF membranes were received preconditioned in a mixture of water and glycerin. These membranes were directly placed in the deposition chamber for the atomic layer deposition (ALD) process. The efficacy of glycerin conditioning for ALD was previously demonstrated in another study [18]. After the deposition process, the membranes were subjected to successive rinses in water baths to remove any remaining glycerin and to determine their functional properties. To initiate the deposition process, the preconditioned PSF membranes were introduced into the home-built ALD reactor and allowed to acclimate for 15 min under vacuum conditions (~10^−2^ mbar). The reactor was preheated to 100 °C. For all deposition runs, we aimed for a 2 nm thickness while guided by the successful outcomes of a prior study involving TiO_2_ deposition on PSF HF membranes [18]. The number of cycles for each oxide was determined theoretically while taking into account the growth rates observed in a previous study [36] to achieve the targeted 2 nm deposition thickness. Specifically, TiO_2_ exhibited a growth rate of 0.1 nm per cycle [18], while both ZnO and Al_2_O_3_ demonstrated a growth rate of 0.2 nm per cycle [36], resulting in the application of 20 and 10 cycles, respectively. The deposition processes utilized specific precursors, and all parameters (including pulse, exposure, and purge times) are detailed in Table 3. The precursors were contained in stainless steel cylinders, and the lines leading to the reactor were heated to 80 °C to prevent any condensation.

### 3.3. Characterizations

The morphology of the membranes, both in cross-section and on the surface, was examined using a Hitachi S4800 scanning electron microscopy system (SEM). Prior to imaging, the membranes underwent nitrogen cold-cutting. Additionally, energy-dispersive X-ray spectroscopy (EDX) analysis was conducted using a Zeiss EVO HD15 microscope equipped with an Oxford X-MaxN EDX detector. To investigate the changes in chemical structure and confirm the successful deposition of oxides on the PSF HF membranes, Fourier transform infrared (FTIR) spectra were acquired. An FTIR spectrometer (NEXUS instrument) equipped with an attenuated total reflection (ATR) accessory was used to obtain spectra in the frequency range of 600–4000 cm^−1^. The ATR-FTIR spectra were recorded at a resolution of 4 cm^−1^, and an average of 64 scans were taken. Moreover, X-ray photoelectron spectroscopy (XPS) was performed using a Kratos AX-IS NOVA spectrometer (Kratos Analytical Ltd., Manchester, UK) equipped with a mono-chromatized Al Kα X-ray source (1486.6 eV) operating at a power of 150 W (10 mA, 15 kV). Survey and high-resolution spectra were obtained using 160 and 20 eV pass energies, respectively. The binding energy (BE) measurements were corrected based on the C1s energy at 284.4 eV. The experiments were conducted on PSF HF segments securely mounted on a support with a primary focus on analyzing the external surface of the membrane. The mechanical properties of both the raw and modified PSF HF membranes were evaluated using a dynamic mechanical analysis device (Z005, 5 kN Proline, Zwick Roell, Ulm, Germany). A 10N sensor and a tensile testing speed of 0.4 mm·min^−1^ were applied to determine the Young’s modulus of the membranes. Water contact angles (WCAs) were measured to assess the hydrophilicity of the membranes. An ILMS GBX tensiometer/goniometer equipped with an optic telecentric F55 double focal monochrome and GBX software (County Dublin D24, Ireland, https://gbxonline.com/ accessed on 10 August 2023) was used for the measurements. Approximately 0.5 μL of ultrapure water was deposited on the membranes using a needle for each sample.

### 3.4. Filtration Experiments

In the experimental setup, a U-tube-shaped hollow fiber bundle was created using a homemade polyvinyl chloride (PVC) housing module. The bundle consisted of 10 wet fibers, each measuring 40 cm in length, resulting in an effective membrane area of 90 ± 5 cm^2^. The module was sealed using epoxy resin. To measure the pure water flux (Jw) for each membrane, pure water was circulated through the membrane system under applied pressures ranging from 0.5 to 1.5 bar. The flow passing through the membrane was measured for 60 s. Jw was calculated using the following formula:

(1) $$\mathrm{Jw}=\frac{Q}{A}\;\left(L\cdot h^{-1}\cdot m^{-2}\right)$$

where Q (L·h^−1^) represents the amount of water that passed through the membrane and A (m^2^) is the membrane geometric area. The permeability was determined by analyzing the linear variation of Jw versus the applied pressure.

For the rejection experiments, BSA was used as a solute. An I-shaped hollow fiber bundle consisting of 10 wet fibers measuring 27 cm in length was prepared using a PVC module and epoxy resin for sealing. The effective calculated membrane geometric area was 63.28 cm^2^. The BSA contents in the feed (C_f_) and permeate (C_p_) solutions were determined using a spectrophotometer. The solute rejection (R) was calculated using the following formula:

(2) $$R=1-\frac{C_{p}}{C_{f}}\times 100\;\left(\%\right)$$

The membrane was first conditioned with distilled water at a pressure of 1.5 bar for 30 min. External–internal solute filtration was then carried out for 20 min at 0.5 bar. To estimate the antifouling properties of the PSF HF membranes, static protein adsorption was performed. A BSA solution was prepared by dissolving 2 g of BSA in 2 L of PBS solution at pH 7.4. The membrane was conditioned by applying a pressure of 1.5 bar with distilled water for 30 min. Initial external–internal water filtration was conducted for 20 min at 0.5 bar, and the steady pure water flux (${\;J}_{w_{0}}$) was recorded.

The tank was then filled with the BSA solution, and filtration was carried out at 0.5 bar for 2 h, during which the foulant flux (${\;J}_{w_{f}}$) was recorded. The BSA was subsequently removed via mechanical agitation and backwashing with water at a pressure of 1.5 bar internally–externally for 5 min. Finally, a last external–internal filtration cycle with water for 20 min at 0.5 bar was performed, and the steady flux ($J_{w_{1}}$) was recorded.

Several ratios were calculated to assess the fouling properties, including the total fouling ratio ($R_{t}$), flux recovery ratio (FRR), reversible fouling ratio ($R_{r}$), and irreversible fouling ratio ($R_{\mathrm{ir}}$), using the following equations [37]:

(3) $$R_{t}=\frac{J_{w_{0}}-J_{w_{f}}}{J_{w_{0}}}\times 100\;\left(\%\right)$$

(4) $$\mathrm{FRR}=\frac{J_{w_{1}}}{J_{w_{0}}}\times 100\;\left(\%\right)$$

(5) $$R_{r}=\frac{J_{w_{1}}-J_{w_{f}}}{J_{w_{0}}}\times 100\;\left(\%\right)$$

(6) $$R_{\mathrm{ir}}=\frac{J_{w_{0}}-J_{w_{1}}}{J_{w_{0}}}\times 100\;\left(\%\right)$$

The feed foulant solution, foulant retentate, and permeate concentrations were calculated using the Beer–Lambert law with a constant value of k = 0.6439 L/g and an ultraviolet (UV) spectrometer for the absorbance at 280 nm (Uviline Connect Series 940). The rejection of the foulant (R) could be determined using Equation (2), where Cp represents the concentration of the foulant in the permeate and Cf represents the concentration of the foulant in the feed. The rejection was calculated as a percentage and represented the percentage of the foulant that was retained or rejected by the membrane during the filtration process.

## 4. Conclusions

Atomic layer deposition of TiO_2_, ZnO, and Al_2_O_3_ on PSF HF membranes was studied. Based on the first study of TiO_2_ deposition on PSF HF membranes, different oxides were tested following a glycerin conditioning to protect the pores during the process time, and a 2 nm deposition thickness was targeted. The presence of each oxide was proved via EDX analysis. SEM and analysis of the mechanical properties allowed us to ensure that the morphology and integrity of the membranes were not affected by the deposition. Hydrophilicity improvement was confirmed by a WCA reduction from 90° for the raw PSF HF membrane to 70° for the TiO_2_ and ZnO oxides and even 46.5° for the Al_2_O_3_-modified PSF HF membranes. All modified membranes exhibited a great water permeability and stable BSA retention rate after deposition, but only the TiO_2_-modified PSF HF membranes showed enhanced fouling parameters with an optimum 100% FRR and a 0% $R_{ir}$. Expanding the study to include the filtration of other molecules such as humic acid or bacteria would provide a more comprehensive understanding of the performance of the modified PSF HF membranes. By considering a broader range of foulants, such a study can provide valuable insights into the versatility and potential applications of the modified PSF HF membranes in diverse water treatment methods and other relevant fields.

## Figures and Tables

**Table 1:** EDX data showing the composition of the raw and modified membranes (the error was calculated based on a 10% EDX measurement uncertainty).

Atomic Percentages (%)					
Membranes	C	O	Ti	Zn	Al
Raw	86.8 ± 8.6	9.9 ± 0.9	/	/	/
TiO_2_	87.2 ± 8.7	9.9 ± 0.9	≤0.1	/	/
ZnO	62.4 ± 6.2	34.3 ± 3.4	/	1.7 ± 0.2	/
Al_2_O_3_	48.3 ± 4.8	47.8 ± 4.8	/	/	3.2 ± 0.3

**Table 2:** Tensile properties of raw and modified HF membranes (the error refers to the standard deviation of 3 samples).

Oxides	Young’s Modulus (MPa)	Strength at Break (N)	Elongation at Break (mm)
Raw	132 ± 5	2.10 ± 0.02	55 ± 5
TiO_2_	143 ± 6	1.84 ± 0.05	62 ± 7
ZnO	157 ± 5	1.31 ± 0.17	49 ± 8
Al_2_O_3_	132 ± 3	1.12 ± 0.05	38 ± 5

**Table 3:** Deposition protocols of modified PSF HF membranes.

Oxide	TiO_2_	ZnO	Al_2_O_3_
Precursor 1	TiCl_4_	DEZ	TMA
Pulse time 1 (s)	0.5	0.4	0.4
Precursor 2	H_2_O	H_2_O	H_2_O
Pulse time 2 (s)	2	2	2
Temperature (°C)	100	100	100
Exposure time for both precursors (s)	10	30	40
Purge time for both precursors (s)	60	40	60
Argon mass flow during purge (sccm)	100	100	100
Number of cycles	20	10	10

## Data Availability

Data is contained within the article or Supplementary Material.

## Supplementary Materials

The following supporting information can be downloaded at: https://www.mdpi.com/article/10.3390/molecules28166133/s1, Figure S1. Raw P and modified P-1 (TiO2), P-2 (ZnO), P-3 (Al2O3), P-4 (alucone) PSF membranes ATR-FTIR spectra. Figure S2. Cross section and top surface primary SEM images of (a) raw, (b) TiO2, (c) ZnO and (d) Al2O3 modified PSF HF membranes. Figure S3. Survey XPS spectra (a) and deconvoluted XPS spectra of raw membrane C 1s (b) and O 1s (c) and 50 TiO2 cycles PSF HF membrane C 1s (d), O 1s (e) and Ti 2p (f). Figure S4. SEM Images of (a) external surface and (b) cross section of PSF HF membrane that underwent same vacuum and temperature conditions but without TiO2 deposition and (c) external surface and (d) cross section of TiO2 ALD modified PSF HF membrane. Table (e) is presenting permeability values of both these membranes. Figure S5. Tensile strength curves of (a) raw, (b) TiO_2_, (c) ZnO and (d) Al_2_O_3_ modified PSF HF membranes
