# Supplementary material for: Enhancing Water Treatment Performance of Porous Polysulfone Hollow Fiber Membranes through Atomic Layer Deposition
Source: Molecules. 2023 Aug 18;28(16):6133. doi: 10.3390/molecules28166133 (PMC10458008; doi:10.3390/molecules28166133)
Supplement: Supplementary file 1 [file molecules-28-06133-s001.zip › molecules-2529353-supplementary.pdf]

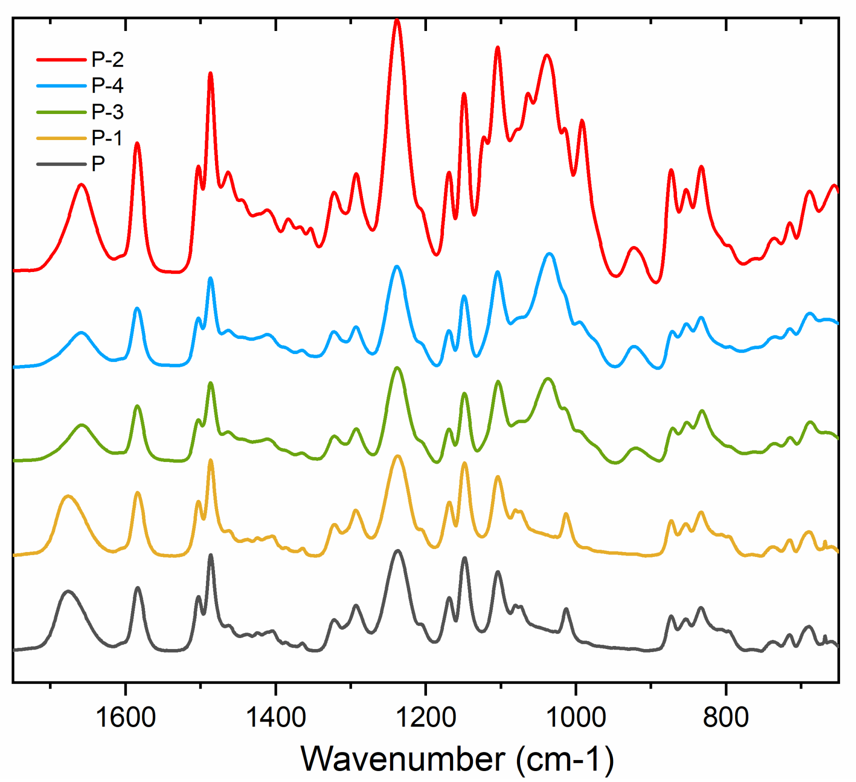

Figure S1. Raw P and modified P-1 (TiO_2), P-2 (ZnO), P-3 (Al_2O_3), P-4 (alucone) PSF membranes ATR-FTIR spectra.

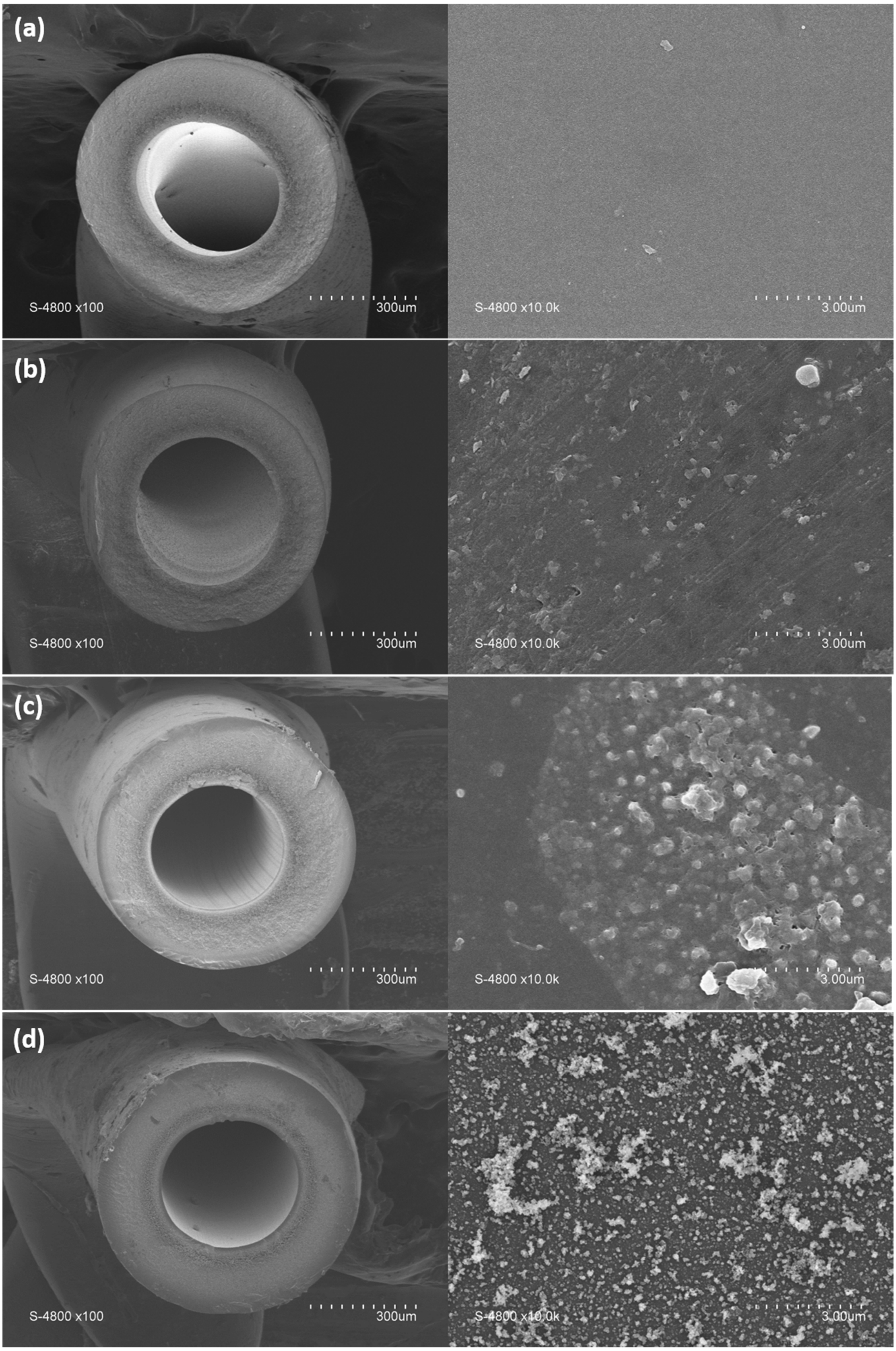

Figure S2. Cross section and top surface primary SEM images of (a) raw, (b) TiO_2, (c) ZnO and (d) Al_2O_3 modified PSF HF membranes.

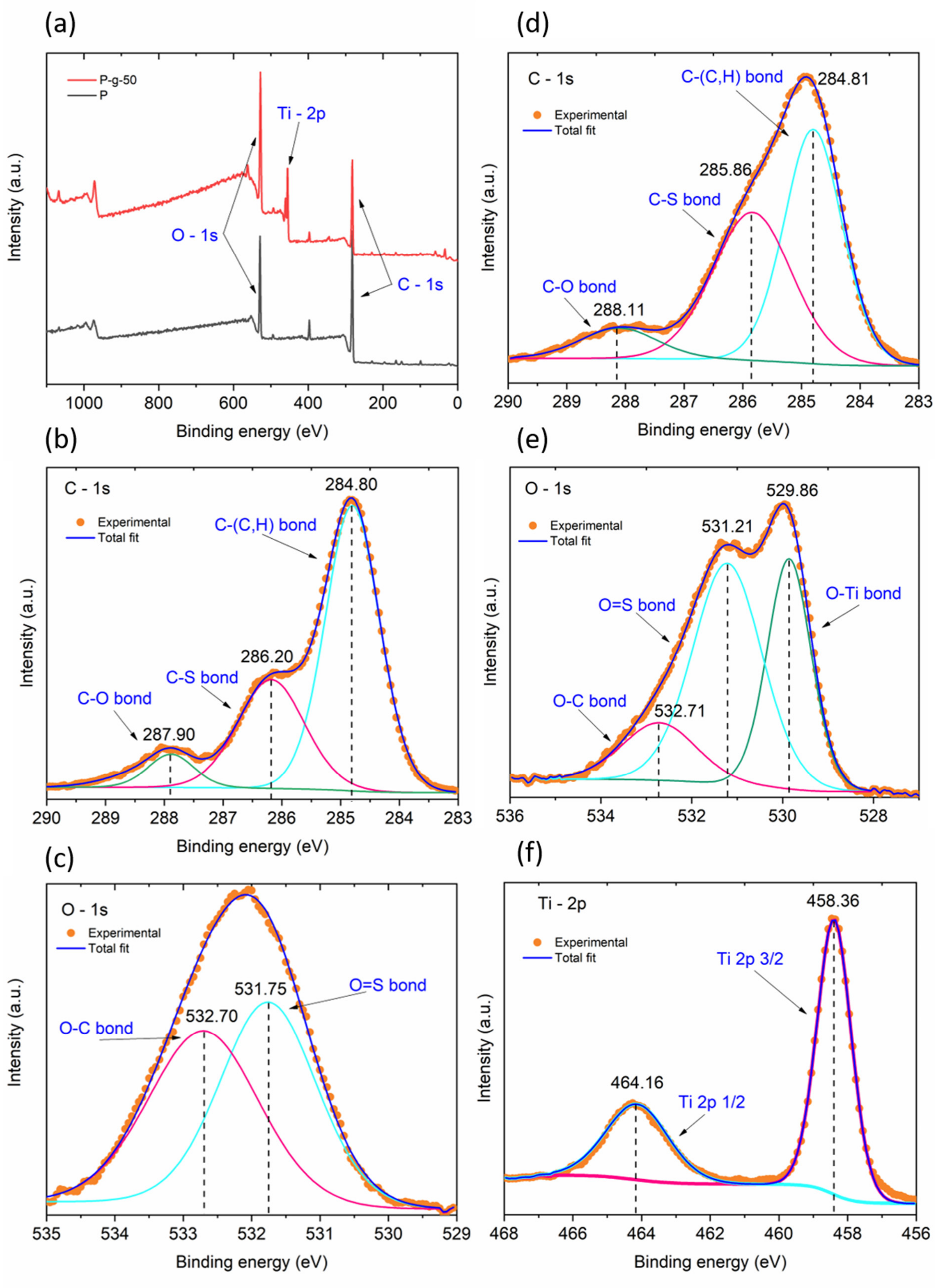

Figure S3. Survey XPS spectra (a) and deconvoluted XPS spectra of raw membrane C 1s (b) and O 1s (c) and 50 TiO_2 cycles PSF HF membrane C 1s (d), O 1s (e) and Ti 2p (f).

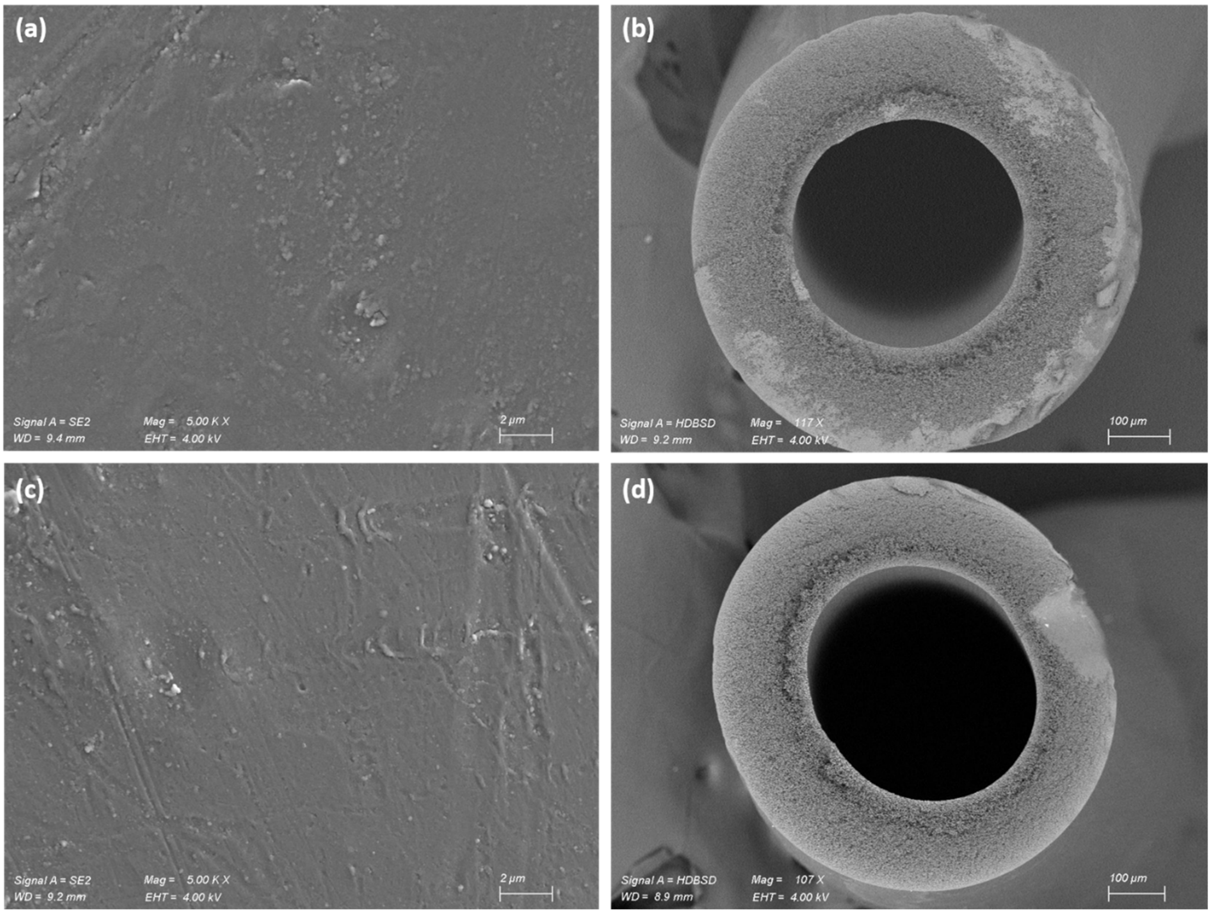

(e)

Treatment	Permeability (L/hm2bar)
Under vacuum and temperature without deposition	27 ± 4
With TiO_2 20 cycles deposition	200 ± 20

Figure S4. SEM Images of (a) external surface and (b) cross section of PSF HF membrane that underwent same vacuum and temperature conditions but without TiO2 deposition and (c) external surface and (d) cross section of TiO2 ALD modified PSF HF membrane. Table (e) is presenting permeability values of both these membranes.

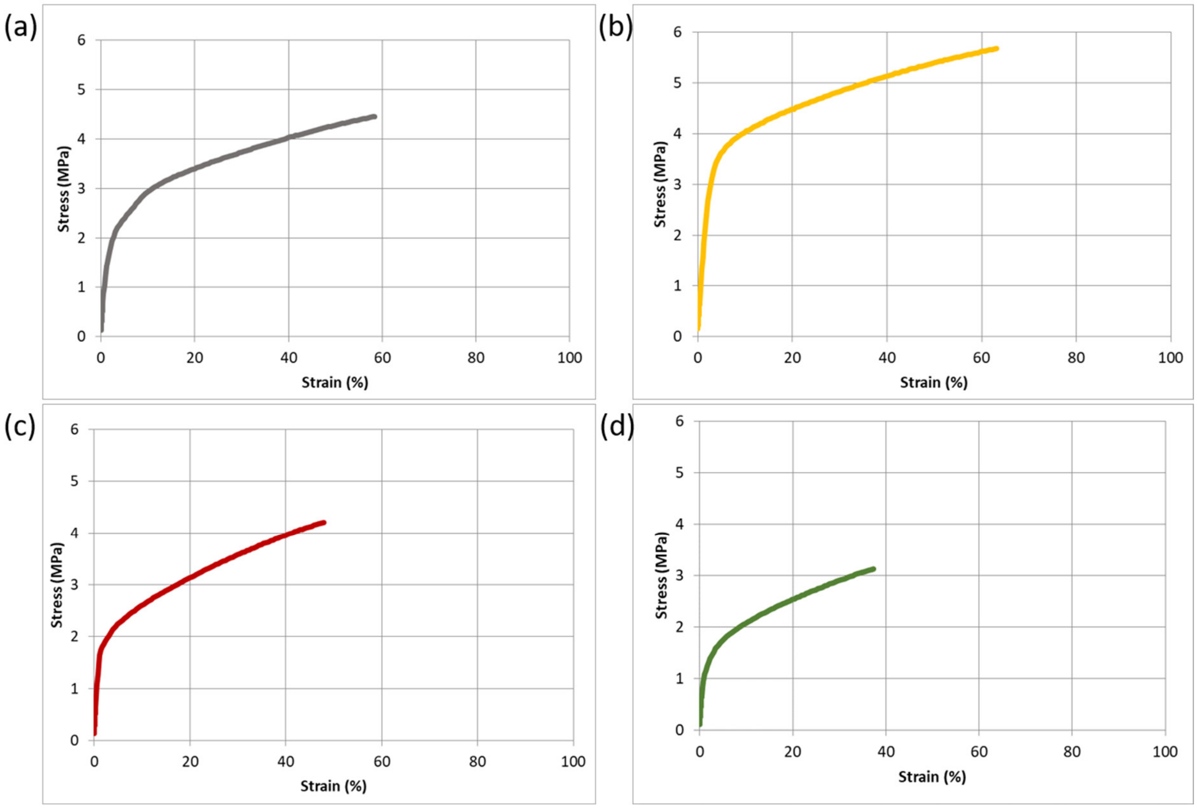

Figure S5. Tensile strength curves of (a) raw, (b) TiO_2, (c) ZnO and (d) Al_2O_3 modified PSF HF membranes.
